# Addition of 2-(ethylamino)acetonitrile group to nitroxoline results in significantly improved anti-tumor activity *in vitro* and *in vivo*

**DOI:** 10.18632/oncotarget.19296

**Published:** 2017-07-17

**Authors:** Ana Mitrović, Izidor Sosič, Špela Kos, Urša Lampreht Tratar, Barbara Breznik, Simona Kranjc, Bojana Mirković, Stanislav Gobec, Tamara Lah, Gregor Serša, Janko Kos

**Affiliations:** ^1^ Faculty of Pharmacy, University of Ljubljana, 1000 Ljubljana, Slovenia; ^2^ Department of Experimental Oncology, Institute of Oncology Ljubljana, 1000 Ljubljana, Slovenia; ^3^ Department of Genetic Toxicology and Cancer Biology, National Institute of Biology, 1000 Ljubljana, Slovenia; ^4^ International Postgraduate School Jožef Stefan, 1000 Ljubljana, Slovenia; ^5^ Department of Biotechnology, Jožef Stefan Institute, 1000 Ljubljana, Slovenia

**Keywords:** nitroxoline derivative, cathepsin B, inhibition, tumor invasion, cell migration

## Abstract

Lysosomal cysteine peptidase cathepsin B, involved in multiple processes associated with tumor progression, is validated as a target for anti-cancer therapy. Nitroxoline, a known antimicrobial agent, is a potent and selective inhibitor of cathepsin B, hence reducing tumor progression *in vitro* and *in vivo*. In order to further improve its anti-cancer properties we developed a number of derivatives using structure-based chemical synthesis. Of these, the 7-aminomethylated derivative (compound **17**) exhibited significantly improved kinetic properties over nitroxoline, inhibiting cathepsin B endopeptidase activity selectively. In the present study, we have evaluated its anti-cancer properties. It was more effective than nitroxoline in reducing tumor cell invasion and migration, as determined *in vitro* on two-dimensional cell models and tumor spheroids, under either endpoint or real time conditions. Moreover, it exhibited improved action over nitroxoline in impairing tumor growth *in vivo* in LPB mouse fibrosarcoma tumors in C57Bl/6 mice. Taken together, the addition of a 2-(ethylamino)acetonitrile group to nitroxoline at position 7 significantly improves its pharmacological characteristics and its potential for use as an anti-cancer drug.

## INTRODUCTION

The proteolytic potential of lysosomal cysteine peptidases is highly relevant for cancer progression, initiating and assisting invasion related proteolytic cascades at the invasive edges of tumors. Of these, cathepsin B (EC 3.4.22.1) has been identified as an important tumor promoting factor [1–3], involved in extracellular matrix (ECM) degradation, a process enabling tumor migration, invasion, metastasis and angiogenesis [1, 4]. Cathepsin B can modulate the ECM, either directly by proteolytic degradation of its components, such as laminin, fibronectin, collagen type I and IV and proteoglycans, or indirectly with the activation of other proteases downstream in a proteolytic cascade [5–9]. Additionally, cathepsin B enhances tumor progression by proteolytic degradation of the metalloprotease inhibitors, TIMP-1 and TIMP-2, and by release of growth factors VEGF and TGF-β1 bound to the ECM proteins [10–12].

Cathepsin B is unique among cysteine cathepsins because it acts as both an endopeptidase and an exopeptidase [13]. Its dual activity is due to the presence of an extra structural element termed the occluding loop [14]. This ~20 amino acid long occluding loop blocks binding of longer endopeptidase substrates, as it covers the S2’ and S3’ subsites of the active site cleft [15]. In the acidic milieu two salt bridges (Asp22-His110 and Asp224-Arg116) bind the loop to the body of the enzyme, limiting access of extended substrates to primed sites of the active site cleft, thereby favoring its dipeptidyl carboxypeptidase activity [14–17]. When the pH increases the salt bridges are disrupted and the resulting conformational change increases the enzyme's endopeptidase activity, with an optimum at neutral pH values [16, 18]. It has been proposed that the endopeptidase activity is involved predominantly in ECM degradation and tumor progression, although recent results suggest also the contribution of exopeptidase activity in tumor progression [19].

Cathepsin B has been validated as a promising target in the treatment of cancer, using a range of research tools, including cathepsin B-specific inhibitors, siRNAs and cathepsin B knock-down or overexpression mouse models [4, 20]. Several groups of cathepsin B inhibitors have been identified. The majority are covalent inhibitors that contain a peptidyl backbone with an electrophilic warhead that reacts with the active site cysteine residue, either reversibly or irreversibly [2, 21, 22]. However, due to their low bioavailability and off-target side effects, none of these compounds can be translated into clinical practice [2]. However, a number of non-covalent, reversible, small molecule inhibitors of cathepsin B have recently been reported [20, 23–27]. We identified nitroxoline (5-nitro-8-hydroxyquinoline), a well-established antimicrobial agent for the treatment of urinary tract infections, as a potent, reversible and selective non-covalent inhibitor of cathepsin B endopeptidase activity [27]. It was shown to reduce significantly ECM degradation, cell proliferation, tumor invasion and endothelial tube formation and induce cell cycle arrest *in vitro* in a number of cell lines. Moreover, it significantly abrogated tumor growth, angiogenesis and metastasis *in vivo* in various mouse models [20, 28–32]. In order to further improve the anti-cancer properties of nitroxoline we designed and synthesized a number of its derivatives [27, 33]. Among them, 2-{[(8-hydroxy-5-nitroquinoline-7-yl)methyl]amino}-acetonitrile (compound **17**) showed significantly improved kinetic properties for inhibition of cathepsin B endopeptidase activity [19, 33].

In the present study, we have further evaluated its effect on tumor growth, invasion and migration *in vitro* and *in vivo*. Compound **17** impairs tumor cell invasion and migration more effectively than nitroxoline, as monitored *in vitro* on two- and three-dimensional (2D and 3D) models, in both endpoint and real time conditions. Moreover, it also delayed the growth of LPB fibrosarcoma tumors in C57Bl/6 mice more strongly than nitroxoline, thus designating compound **17** as a promising candidate for evaluation of its potential in anti-cancer therapy.

## RESULTS

### Compound 17 impairs tumor cell invasion

**Figure 1:**
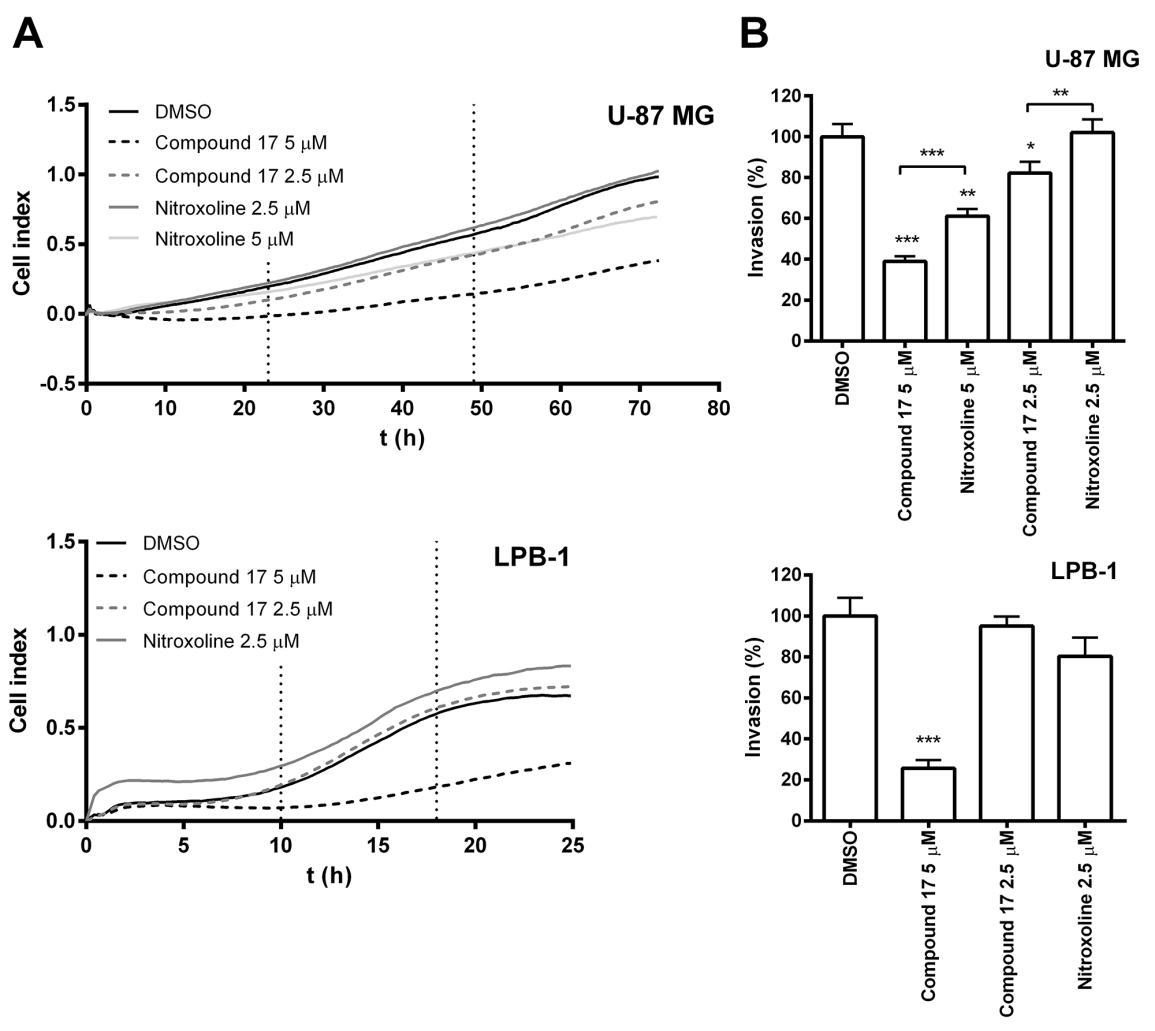
Compound 17 impairs the invasion of tumor cells **(A)** Tumor cell invasion monitored in real time. Upper compartments of CIM-plate 16 were coated with Matrigel (2 mg/mL and 1 mg/ml for U-87 MG and LPB-1 cells, respectively). U-87 MG (7.5 × 10^4^) or LPB-1 (5 × 10^4^) cells were then seeded on top of it. The growth medium in the upper and lower compartments of the CIM-plate 16 was supplemented with compound **17** (2.5 μM or 5 μM), nitroxoline (2.5 μM or 5 μM) or DMSO (0.05%) as a control. Tumor cell invasion was then monitored continuously for 72 h by measuring impedance (reported as CI) using the xCELLigence system. **(B)** The ability of the cells to invade correlated to the slopes (1/h) in the time interval between 23 and 49 h for U-87 MG cells and between 10 and 18 h for LPB-1 cells and was used to calculate the percentage of invasion (%), presented as means ± SEM. The experiments were performed in quadruplicate and repeated three times. * *P* < 0.05, ** *P* < 0.01, *** *P* < 0.001.

The ability of compound **17** to reduce tumor cell invasion was evaluated on the human glioma cell line U-87 MG and on the mouse fibrosarcoma cell line LPB-1. Invasion was monitored in real time using the xCELLigence system [34]. This system measures invasion of cells through Matrigel, a model of ECM, by monitoring the impedance, expressed as cell index (CI) (Figure 1A), across microelectrodes integrated in the membrane between top and bottom compartments of the CIM (cell invasion and migration)-plate 16. This was carried out over the entire course of the experiment. Compound **17** significantly reduced invasion of tumor cell lines, at 2.5 μM concentration for U-87 MG cells by 21 ± 5% and at 5 μM concentration by 61 ± 3% and 74 ± 4% for U-87 MG and LPB-1 cells (Figure 1B). Furthermore, it shows improved inhibition of tumor invasion on U-87 MG cells compared to nitroxoline.

**Figure 2:**
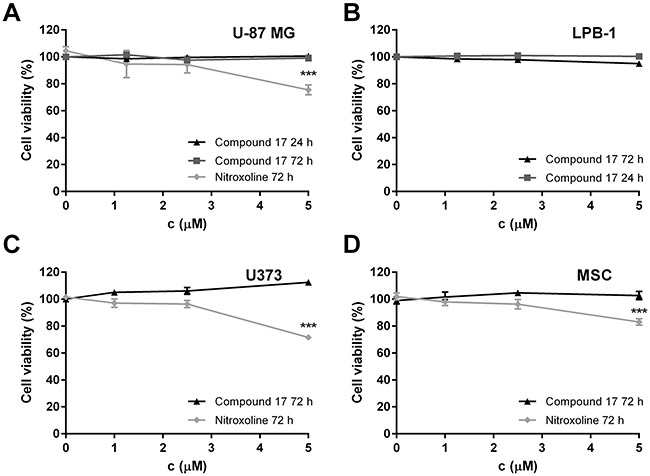
The cytotoxicity of compound 17 on U-87 MG, U373 and LPB-1 cells and mesenchymal stem cells (MCS) as determined by MTS assay **(A)** U-87 MG cells (3 × 10^4^ and 5 × 10^3^ for 24 and 72 h, respectively), **(B)** LPB-1 cells (1 × 10^5^ and 2.5 × 10^3^ for 24 and 72 h, respectively), **(C)** U373 cells (3 × 10^4^) and **(D)** MSCs (3 × 10^4^) treated with increasing concentrations of compound **17** and nitroxoline for 24 or 72 h, following addition of MTS reagent. Results are presented as the percentage of viable cells from two independent experiments (mean ± SEM) in the presence of the inhibitor compared to DMSO used as a control. The experiments were performed in quadruplicate. ***P < 0.001.

To exclude the possibility that the reduction of tumor cell invasion was due to compound **17**-induced cytotoxicity, its effect on cell viability was evaluated by MTS cell viability assay. After treatment with compound **17** at concentrations up to 5 μM for 24 or 72 h, the viability of neither cell line was reduced (Figure 2). On the other hand, nitroxoline did not affect cell viability of U-87 MG cells at concentrations up to only 2.5 μM (Figure 2), however it did not affect cell viability of LPB-1 cells in concentration up to 5 μM [20].

### Compound 17 reduces tumor cell invasion in a three-dimensional assay

**Figure 3:**
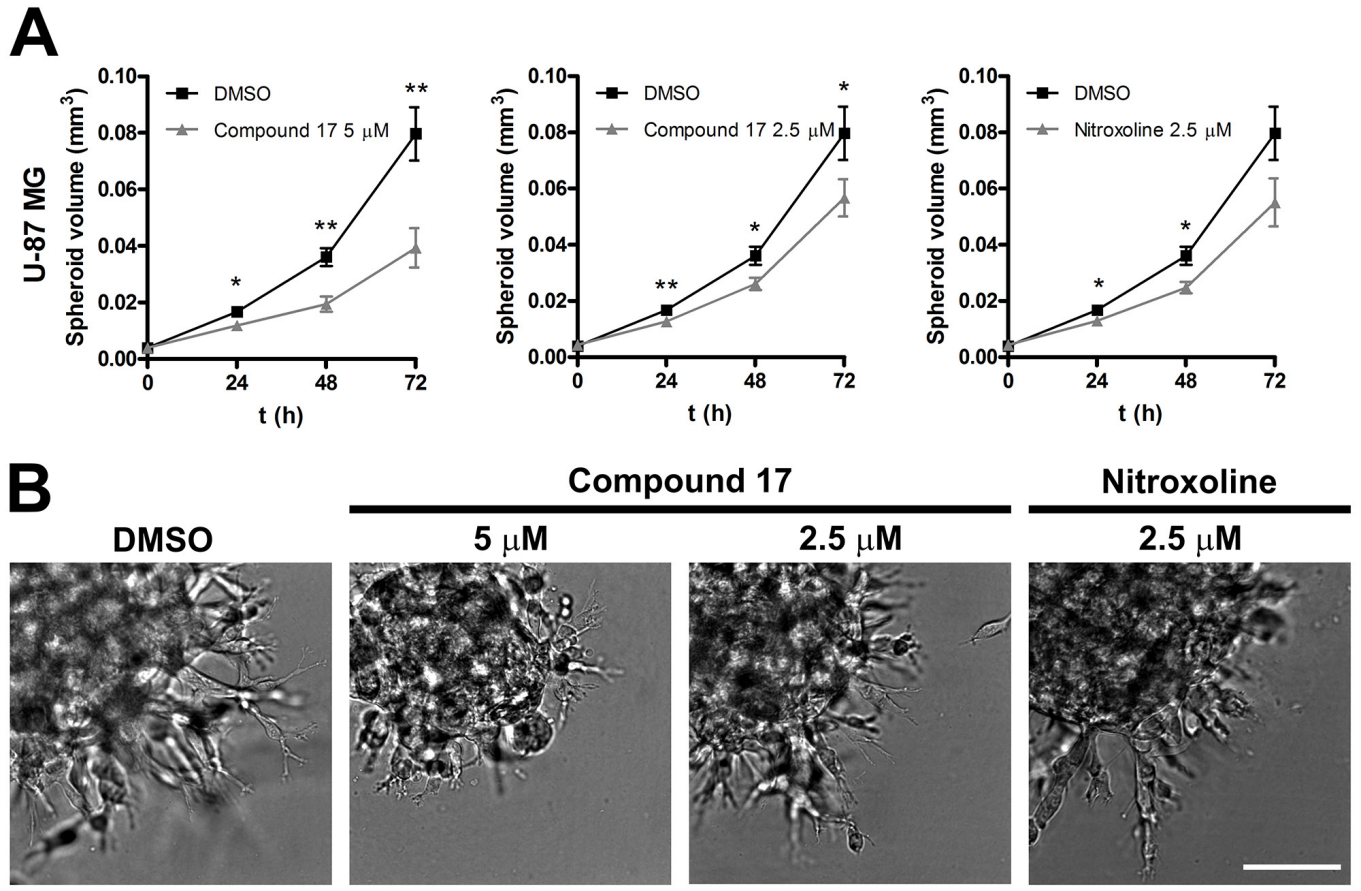
Compound 17 impairs invasion of U-87 MG cells in a 3D *in vitro* model of tumor invasion **(A)** U-87 MG spheroids were implanted in Matrigel (5 mg/mL) and covered with growth medium. Compound **17** (2.5 μM or 5 μM), nitroxoline (2.5 μM) or DMSO (0.05%) as a control were added to the growth medium and to Matrigel. The spheroid volume was monitored for up to three days by measuring the spheroid dimensions. Data are presented as means ± SEM (n = 3). **(B)** Representative images of U-87 MG spheroids obtained at day three after implantation. Scale bar, 100 μm. * *P* < 0.05, ** *P* < 0.01.

Compound **17** was further evaluated for its ability to impair tumor cell invasion using a 3D *in vitro* tumor cell invasion model. This model is based on implantation of tumor spheroids into ECM-mimicking matrices and, as such, mimics early, avascular stages of tumor growth. It is therefore a more representative model of tumor cell invasion than are classic models involving cell monolayers. Spheroids from U-87 MG cells were prepared using the hanging drop method [35] following their implantation in Matrigel. Growth of spheroids was monitored for three days by measuring their dimensions under a light microscope with an ocular micrometer (Figure 3A). Representative images of U-87 MG spheroids were recorded on day three after implantation. U-87 MG spheroids displayed steady growth and formed invasive stands in a sunburst pattern around the original spheroid during the entire course of the experiment (Figure 3B). Incubation of U-87 MG spheroids in the presence of compound **17** (2.5 and 5 μM) and of nitroxoline (2.5 μM) significantly reduced the growth of spheroids, the effect being more pronounced at the higher concentration of compound **17** (51 ± 9%).

### Compound 17 inhibits cell migration in a three-dimensional assay

**Figure 4:**
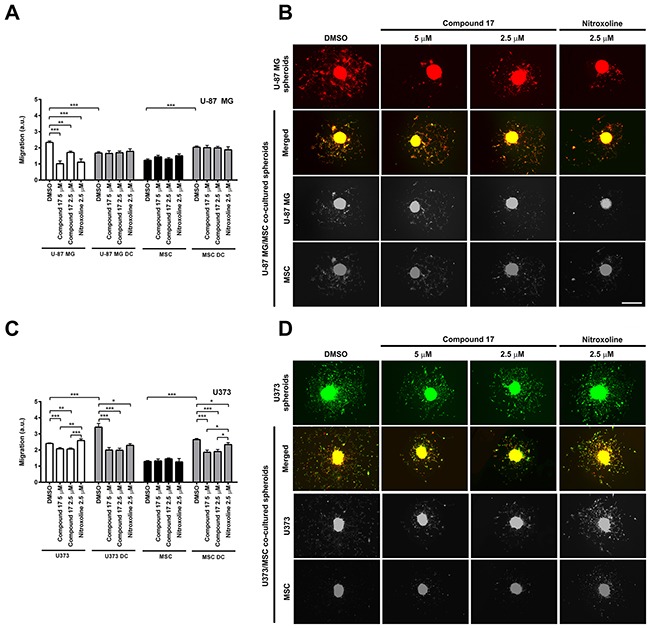
Effects of compound 17 and nitroxoline on the migration of cells out of co-cultured spheroids **(A and C)** U-87 MG, U373 or MSC spheroids or MSC/glioblastoma direct co-cultured (DC) spheroids were, after generation, placed in the middle of laminin-coated wells of 96-well plates in medium containing compound **17** (2.5 μM or 5 μM), nitroxoline (2.5 μM) or DMSO (0.1%) as a suitable control. Migration of cells was expressed as relative migration and was assessed on day three. Relative migration was calculated as the ratio of migration distance to spheroid diameter, of MSCs and U-87 MG or U373 cells, respectively, out of monoculture and co-cultured spheroids in presence of compound **17** (2.5 μM or 5 μM), nitroxoline (2.5 μM) or control after 72 h on laminin. Experiments were performed in triplicate and are presented as means ± SEM. **(B and D)** Representative images of cell migration out of spheroids, obtained after 72 h on laminin. U-87 MG cells are red; U373 cells are green. MSCs in U-87 MG/MSC spheroids are green and in U373/MSC spheroids are red. Single-channel images from co-cultured spheroids are displayed in grayscale. Scale bar, 500 μm. Statistical analysis was performed with one-way ANOVA, followed by the Bonferroni post-hoc test. *P < 0.05, **P < 0.01, ***P < 0.001.

Compound **17** was evaluated for its ability to inhibit migration of two glioblastoma cell lines, U-87 MG and U373 cells out of spheroids. Relative migration, calculated as the ratio between migration distance and spheroid diameter, was assessed on day three after the generation of spheroids. Compound **17** (2.5 and 5 μM) and nitroxoline (2.5 μM) both significantly decreased migration of cells out of U-87 MG spheroids, by 24 ± 3% and 61 ± 6% at 2.5 and 5 μM concentrations of compound **17**, and by 52 ± 8% for nitroxoline (2.5 μM; Figure 4A and 4B). However, only compound **17** inhibited migration of cells out of U373 spheroids by 14 ± 3 % at both concentrations used (Figure 4C and 4D). Its effect on the migration of U-87 MG or U373 cells and mesenchymal stem cells (MSCs) out of co-cultured spheroids was also monitored. Co-cultured spheroids constitute a more complex model that takes into account interactions of tumor cells with stromal cells derived from the tumor microenvironment. In U-87 MG/MSC co-cultured spheroids, the migration of U-87 MG cells decreased compared to U-87 MG monoculture spheroids, while the migration of MSCs increased (Figure 4B). Neither compound **17** (2.5 and 5 μM) nor nitroxoline (2.5 μM) had any effect on cell migration out of U-87 MG/MSC co-cultured spheroids (Figure 4A). Interactions between MSCs and U373 cells, on the other hand, increased migration out of U373/MSC co-cultured spheroids of both cell lines, U373 and MSCs, (Figure 4C). Interestingly, after treatment with compound **17** or nitroxoline a significant decrease in cell migration out of U373/MSC co-cultured spheroids was observed for both cell lines compared to that for control cells treated with DMSO. Compound **17** decreased migration of U373 cells by 42 ± 4% and 42 ± 5% at 2.5 and 5 μM concentrations respectively, and nitroxoline by 33 ± 3%. Simultaneously, MSC cell migration was decreased by 28 ± 5% and 30 ± 5% at 2.5 and 5 μM concentrations of compound **17**, and by 12 ± 5% for nitroxoline (2.5 μM; Figure 4C).

Compound **17** did not induce cytotoxicity of U-87 MG, U373 cells or MSC in concentrations up to 5 μM, as evaluated by MTS assay, while nitroxoline was not cytotoxic at concentrations up to only 2.5 μM (Figure 2).

### Compound 17 delayed tumor growth *in vivo*

**Figure 5:**
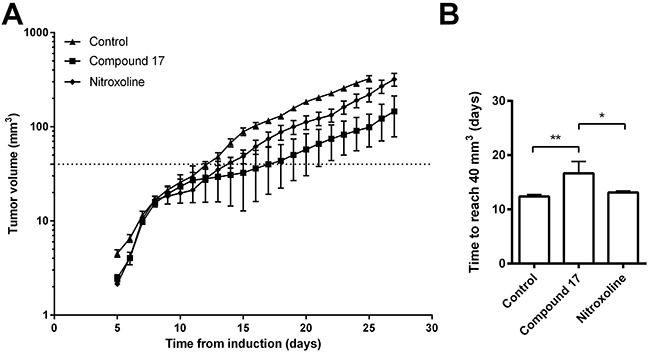
Compound 17 reduces tumor growth in an LPB mouse tumor model **(A)** LPB cells (1.8 × 10^6^) were injected into the right flank of C57BI/6 mice following treatment with compound **17** or nitroxoline at 20 mg/kg, with drinking water *ad libitum* or, in the case of the control group, with drinking water only for the entire duration of the experiment. The size of LPB-induced tumors was measured every second day. The line indicates a tumor volume of 40 mm^3^. **(B)** The time required for tumors to reach a volume of 40 mm^3^ representing the tumor growth delay between the experimental and control group, was calculated from the growth curves. Data are presented as means ± SEM. *P < 0.05, **P < 0.01 as determined with one-way ANOVA.

Administration of compound **17** *per os* delayed LPB fibrosarcoma tumor growth *in vivo* in C57Bl/6 mice. Compound **17** (20 mg/kg) and nitroxoline (20 mg/kg) were administered in drinking water for the entire duration of the experiment. Tumor volumes in mice receiving compound **17** or nitroxoline were both smaller in mice receiving drinking water only (Figure 5A). The time tumors needed to reach 40 mm^3^ was significantly increased by compound **17**, but only moderately by nitroxoline treatment (Figure 5B). Similar results were observed for delay of tumor growth, where compound **17** was more effective than nitroxoline (Figure 5B).

Neither compound **17** nor nitroxoline induced any systemic toxicity, as judged by monitoring body weight, behavior and appearance of the mice over the entire course of the experiment.

## DISCUSSION

**Figure 6:**
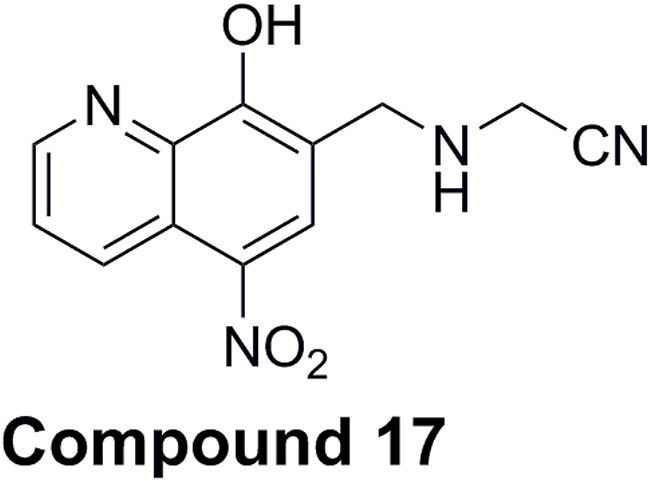
Chemical structure of 2-{[(8-hydroxy-5-nitroquinoline-7-yl)methyl]amino}-acetonitrile (compound 17)

Compound **17** is a product of focused chemical synthesis of nitroxoline derivatives that are based on the crystal structure of nitroxoline-cathepsin B complex [27, 33]. It is nitrile inhibitor that combines nitroxoline scaffold with nitrile fragment connected through amine spacer on position 7 (Figure 6) structure element described as suitable for cysteine cathepsin inhibitors [36]. Due to its potent but reversible inhibition of cathepsin B endopeptidase activity, together with the improved selectivity for cathepsin B with regard to related peptidases cathepsins H and L [33], compound **17** was chosen for further evaluation of its anti-tumor activity *in vitro* and *in vivo*. Compound **17** was shown to reduce tumor cell migration and invasion and tumor growth significantly better than the parent compound nitroxoline.

Cathepsin B is an important tumor promoting enzyme. Its increased expression and activity are associated with various cancer types [37, 38]. Accordingly, to study the effect of compound **17** on tumor cell migration and invasion *in vitro*, we selected cell lines of different origins, all expressing high levels of cathepsin B [20, 39]. Compound **17** was effective in inhibiting tumor cell invasion of the human glioblastoma cells U-87 MG and mouse fibrosarcoma LPB-1 cells (Figure 1). The effect, observed by monitoring tumor invasion in real time on an xCELLigence system, was very pronounced and more reliable than using the end-point approach. Next, compound **17** was evaluated in a 3D invasion assay *in vitro* which, more accurately than the 2D model, reproduces the tumor microenvironment and captures the complexity of tumors [35]. In this assay, both compound **17** and nitroxoline reduced the growth of U-87 MG spheroids in Matrigel (Figure 3). The reason that the effect of both compounds is stronger in a 3D than in a 2D invasion model is that the former is more complex and better resembles the *in vivo* situation. The function of cathepsin B is thus more significant, since it could also participate in the degradation of ECM in cell adhesion and signaling [40], these processes being important for cell organization within the spheroids. Taken together, the presented data from independent models of tumor cell invasion show that compound **17**, by inhibiting cathepsin B endopeptidase activity, acts as a potent inhibitor of tumor cell invasion *in vitro*.

Another process that is enhanced during tumor progression is cell migration [38]. Therefore, in addition to the growth of spheroids and formation of their invading stands in Matrigel, we evaluated the effects of compound **17** and of nitroxoline on the migration of cells out of spheroids, using two human glioblastoma cell lines, U-87 MG and U373. Both compound **17** and nitroxoline significantly decreased migration of cells out of U-87 MG spheroids, while only compound **17** impaired cell migration out of U373 spheroids (Figure 4). In addition to tumor cells, stromal cells, including mesenchymal stem cells (MSCs), also form a tumor microenvironment and their interactions with tumor cells play a crucial role in tumor progression [41–45]. In the presence of glioblastoma cells, MSCs enhance tumor cell invasion through multiple distant processes, among them peptidase activity regulation [46]. We therefore evaluated the effect of cathepsin B inhibitors, compound **17** and nitroxoline on the migration of tumor cells out of co-cultured glioblastoma-MSCs spheroids. In U-87 MG/MSC co-cultured spheroids we observed decreased migration of U-87 MG cells out of spheroids and increased MSC migration. However, the addition of either compound **17** or nitroxoline had no effect on the migration of either U-87 MG cells or MSCs out of co-cultured spheroids. On the other hand, when U373/MSC co-cultured spheroids were prepared and studied, the migration of all cells increased and was significantly impaired by compound **17** as well as by nitroxoline. As in U373 monoculture spheroids, compound **17** exhibited a more pronounced inhibition of cell migration than nitroxoline (Figure 4). Our results are in line with the upregulated cathepsin B levels in glioblastoma U373 cells on direct cell-to-cell interactions with MSCs [45, 47]. Besides expression, cathepsin B secretion was also increased in U373/MSC co-cultured spheroids. Moreover, MSC-mediated invasion of U373 cells decreased after treatment by CA-074Me, a cell permeable analogue of the irreversible cathepsin B inhibitor, CA-074. In contrast, the expression of cathepsin B was not altered in co-cultures of MSCs and other glioblastoma cell line U-87 MG, when compared to U-87 MG monocultures [47].

The anti-cancer properties of compound **17** were further evaluated *in vivo* in an LPB mouse fibrosarcoma tumor model. *Per os* administration of compound **17** reduced the tumor volume and delayed tumor growth (Figure 5). Again, it displayed higher anti-tumor activity than that induced by nitroxoline, since its administration resulted in a significantly greater reduction of tumor volume with prolonged growth delay. Nevertheless, for better assessment of the impact of compound **17** on tumor growth, the concentration of nitroxoline used in this study was just half that used previously [20]. At a higher concentration therefore, it would result in a reduction of tumor growth similar to that in our previous study [20].

Compound **17** is thus shown to be a potent and selective inhibitor of cathepsin B endopeptidase activity and to impair tumor invasion and migration in *in vitro* models, as well as delaying tumor growth in an *in vivo* mouse model. Using focused chemical synthesis, therefore, we have increased the anti-tumor potential of nitroxoline and a novel inhibitor prepared showing more appropriate pharmacological characteristics, making it a promising candidate for further evaluation as an anti-cancer drug.

## MATERIALS AND METHODS

### Compounds

Compound **17** was synthesized as described [33]. Its purity was determined by ^1^H NMR and ^13^C NMR spectroscopy and by CHN elemental analysis. Nitroxoline was obtained from Sigma-Aldrich (St. Louis, MO, USA).

### Cell culture

The human glioblastoma cell lines U-87 MG and U373 were obtained from American Type Culture Collection (ATCC, Manassas, VA, USA). The cell lines were authenticated by ATCC and thawed from early passage stocks. The LPB-1 fibrosarcoma cell line [48], a clonal derivative of the methylcholanthrene-induced C57Bl/6 mouse sarcoma tumor, was provided by Jean Jr. Belehradek (Institut Gustave Roussy, Villejuif, France). Human bone marrow-derived MSCs were obtained from Lonza Bioscience (Lot. 6F4393, Walkersville, MD, USA). All cell lines were passaged for fewer than 6 months after thawing. No authentication was done by the authors. U-87 MG cells were cultured in Advanced Dulbecco's Modified Eagle's Medium (DMEM; Gibco, Carlsbad, CA, USA) supplemented with 10% fetal bovine serum (FBS; Gibco), 2 mM glutamine (Sigma-Aldrich) and antibiotics. U373 cells and MSCs were cultured in DMEM supplemented with 10% FBS, 2 mM glutamine, antibiotics, 1 mM Na-pyruvate (Gibco) and non-essential amino acids (Sigma-Aldrich). LPB-1 cells were cultured in Minimum Essential Medium (MEM; Sigma-Aldrich) supplemented with 10% FBS, 2 mM glutamine and antibiotics. U373 cells stably expressing enhanced green fluorescent protein (U373 eGFP cells) and U87 cells expressing red fluorescent protein (dsRED) were prepared as described [45, 49]. The cells were maintained at 37 °C in a humidified atmosphere containing 5% CO_2_ until 80% confluent. Prior to their use in the assay, cells were detached from the culture flask using 0.05% trypsin (Gibco) and 0.02% EDTA in phosphate buffered saline (PBS), pH = 7.4.

### Cell viability assay

The possible cytotoxic effects of compound **17** and nitroxoline in the cell lines used were determined by MTS [3-(4,5-dimethylthiazol-2-yl)-5-(3-carboxymetoxyphenyl)-2-(4-sulfophenyl)-2H-tetrazolium] colorimetric assay. U-87 MG (3 × 10^4^ and 5 × 10^3^ for 24 and 72 hours respectively), LPB-1 (1 × 10^5^ and 2.5 × 10^3^ for 24 and 72 h, respectively), U373 (3 × 10^4^ cells for 72 h) cells and MSCs (3 × 10^4^ for 72 h) were seeded into wells of a 96-well microplate and allowed to attach overnight. They were then treated with 100 μl of medium containing 1.25, 2.5 or 5 μM of inhibitor or DMSO (0.05%) and incubated for 24 or 72 h. 10 μl of MTS (Promega, Madison, WI, USA) was then added to the walls of a 96-well microplate and, after incubation, absorbance of formazan was measured at 490 nm on a Tecan Safire^2^™ (Tecan, Mannedorf, Switzerland). Cell viability (%) was expressed as the ratio of absorbance obtained in the presence of compounds to that in DMSO alone. All assays were performed in quadruplicate and repeated twice.

### Real-time invasion assay

Tumor cell invasion in real time of U-87 MG and LPB-1 cells was monitored using an xCELLigence RTCA instrument (ACEA Biosciences Inc., San Diego, CA, USA). Before the experiment, the cells were starved of serum for 24 h. First, the bottoms of the wells of a CIM-plate 16 (ACEA Biosciences Inc.) were coated for 30 min with 0.3 μg of fibronectin from bovine plasma (Calbiochem, Darmstadt, Germany). Following coating of the upper compartments of a CIM-plate 16 with 20 μl of Matrigel (2 mg/ml and 1 mg/ml for U-87 MG and LPB-1 cells (BD Biosciences, Franklin Lakes, NJ, USA)) in serum-free medium (SFM). Matrigel was allowed to gel for 20 min at 37 °C. 180 μl of medium containing respective compound (2.5 or 5 μM) or DMSO (0.05 %) as control was added to the lower compartments and the top and the bottom parts of the CIM-plate 16 were assembled together. Next, 60 μl of SFM, together with the compounds to be tested, were added to the upper compartments. After 1 h incubation at 37°C, 80 μl of U-87 MG or LPB-1 cells (7.5 × 10^4^ or 5 × 10^4^ cells/well for U-87 MG or LPB-1 cells) were seeded in the top chambers of a CIM-plate 16 and placed into the xCELLigence system. The system measures impedance data, reported as cell index (CI), in real time every 15 min during the 72 h course of the experiment. The data were analyzed with the RTCA Software (Roche). The relative invasion (%) was expressed as a percentage of that of control cells treated with DMSO.

### Three-dimensional invasion assay

The 3D invasion model is based on implantation of tumor spheroids into Matrigel, representing a model of the ECM (tumor microenvironment). Spheroids were prepared according to the hanging-drop method [35]. Drops (20 μl) of U-87 MG cell suspension (150 cells/drop) were placed on the lids of 100 mm tissue-culture dishes which were then inverted over 10 ml of water. After 5 days, the aggregates formed were transferred to wells of a Lab-Tek™ Chambered Coverglass coated with 70 μl of Matrigel (5 mg/ml) in SFM. Next, a further 70 μl of Matrigel was added to cover the spheroids and, after 20 min incubation at 37°C, 400 μl of complete medium was added. Compounds (2.5 or 5 μM) or DMSO (0.05 %) were added to the Matrigel and the medium. The growth of spheroids was monitored daily for up to three days by measuring the spheroid dimensions under a light microscope, using an ocular micrometer. Spheroid volume was calculated according to the equation: *V = (π × (spheroid length) × (spheroid width)^2^)/6*. Images of tumor spheroids were obtained using an Olympus IX 81 motorized inverted microscope and Cell^R software (Olympus, Tokyo, Japan).

### Migration assay

Cell migration was monitored using fluorescent glioblastoma cells U-87 MG dsRED and U373 eGFP, and MSCs. For separate monitoring of MSCs and glioblastoma cell migration in co-cultured MSC/U-87 MG and MSC/U373 cell spheroids, MSCs were labelled, prior to spheroid formation, with the non-toxic fluorescent dyes Vybrant DiO and Vybrant DiI (Molecular Probes, Eugene, OR, USA) according to the manufacturer's instructions. For co-cultured spheroid formation fluorescent glioblastoma cells and Vybrant-labelled MSCs were mixed together in a 1:1 ratio and seeded, at a density of 2.5 × 10^3^ cells/100 μl in complete growth medium containing 4% methylcellulose, into BD Falcon U-bottom 96-well cell culture plates (BD Biosciences, Franklin Lakes, NJ, USA). They were then centrifuged at 850 x g and 31°C for 90 min and then incubated overnight. For the migration assay, 96-well plates (Corning, Corning, NY, USA) were coated with laminin (2 μg/cm^2^, Sigma-Aldrich) for 2 h at 37°C and washed twice with PBS. U-87 MG, U373 and MSC spheroids from monoculture and co-cultured spheroids were then placed in the middle of each well in 100 μL of growth medium. Compounds (2.5 or 5 μM) or DMSO (0.1%) were added to the culture medium. The cell migration was monitored for up to 3 days under a Nikon Eclipse Ti-E fluorescence inverted microscope (40× magnification, Nikon, Tokyo, Japan). The migration ability of the cells (relative migration) was assessed on day 3 and defined as the distance measured from the edge of the spheroid to the most distant cell population divided by the spheroid diameter.

### Mouse tumor models

#### Ethics statement

Animal studies were carried out in accordance with EU guidelines and with permission from the Veterinary Administration of the Ministry of Agriculture, Forestry and Food of the Republic of Slovenia (permission number: U34401-14/2014/4). Mice were housed in a specific-pathogen-free animal colony at controlled temperature and humidity with a 12 h light/dark cycle. Food and water were provided *ad libitum*.

### LPB mouse fibrosarcoma tumor model

The effect of compounds on tumor growth *in vivo* was observed in C57Bl/6 female mice, 8 to 12 weeks old. 1.8×10^6^ LPB fibrosarcoma cells, in 100 μL of 0.9% NaCl, were injected subcutaneously into the right flank of each mouse. In the experimental group, mice were given a solution of compound **17** (n = 7, 20 mg/kg) or nitroxoline (n = 12, 20 mg/kg – half the dose used in the previous study [20]) in their drinking water *ad libitum* for the entire duration of the study (27 days). The bottle with fresh solution of the compounds was provided every fourth day. The control group (n = 11) received drinking water only. The dose of inhibitor received was calculated from the volume of the consumed solution per mouse provided prior the beginning of the study (2.8 ± 0.1 ml). Tumor growth was determined every 2 to 3 days from the fifth day after the induction of tumors, using a digital Vernier caliper. The tumors were measured in three perpendicular directions (a, b, c) and tumor volume was calculated as *V = a x b x c x Π/6*. The tumor growth curves were used to determine the time required to reach a volume of 40 mm^3^. Tumor growth delay was determined as the difference between the time required for tumors to reach a volume of 40 mm^3^ in the experimental and in the control groups. The weight, behavior and appearance of the mice, using a scoring system, were followed as a general indicator of systemic toxicity.

### Statistical analysis

The GraphPad Prism 6.0 software package was used for data analysis. Data are presented as means ± SEM unless stated otherwise. The statistical significance of the differences between groups of data was evaluated using nonparametric, two-tailed Student's t test, unless stated otherwise. Differences were considered significant at *P* ≤ 0.05.

## Abbreviations

CI: cell index
DMSO: dimethyl sulfoxide
ECM: extracellular matrix
MSC: mesenchymal stem cell
MTS: [3-(4,5-dimethylthiazol-2-yl)-5-(3-carboxymetoxyphenyl)-2-(4-sulfophenyl)-2H-tetrazolium]
2D: two-dimensional
3D: three-dimensional
